# Severe aortic stenosis with high valvulo-arterial impedance (Zva) has more adverse cardiac changes on cardiovascular magnetic resonance

**DOI:** 10.1186/1532-429X-18-S1-P339

**Published:** 2016-01-27

**Authors:** Willis Lam, Francois Pontana, Vassilis Vassiliou, Sanjay Prasad

**Affiliations:** 1 CMR Unit, Royal Brompton Hospital, London, United Kingdom; 2 CHRU de Lille, Lille, France

## Background

The most important challenge in asymptomatic severe aortic stenosis (AS) is the timing for intervention. Valvulo-arterial impedance (Zva), an overall after-load assessment, was shown to be one of the most comprehensive indexes to assess AS. This study aims to evaluate the impacts of Zva on cardiac MRI (CMR) parameters.

## Methods

Patients with severe AS had both pre-operative CMR and trans-thoracic echocardiography (TTE) within one week apart. From TTE, on top of the routine measurements, Zva was calculated as the sum of systolic blood pressure and the aortic valve continuous-wave Doppler mean gradient divided by the left ventricular stroke volume index. Patients were categorized into 2 groups according to their calculated Zva: (a) High Zva (≥4.5 mmHg/mL/m²), and (b) low Zva (<4.5). CMR parameters, namely left ventricle mass index (LVMI), ejection fraction (LVEF), end-diastolic volume (LVEDV), right ventricle end-diastolic volume (RVEDV) and left atrium volume index (LAVI), were compared between the 2 groups.

## Results

36 patients were recruited into the final analysis. They were categorized into 2 groups, high Zva (n = 16) and low Zva (n = 20). Baseline characteristics in both groups were comparable except patients in high Zva group had significantly higher systolic blood pressure (p = 0.026). Both aortic valve area (AVA) by continuity equation on echo and by direct planimetry on CMR were similar in both groups (p = 0.91 and 0.295 respectively). Patients with high Zva had higher LVMI (p = 0.03), lower LVEF (p = 0.04), higher LVEDV (p = 0.03) and RVEDV (p = 0.04), and higher LAVI (p = 0.04).

## Conclusions

In setting of severe aortic stenosis, despite tight aortic valve areas, patients with high valvulo-arterial impedance have worse cardiac parametes and functions on CMR than those with low valvulo-arterial impedence.

**Table 1:** TTE and CMR parameters between high and low Zva groups

	Low Zva (n = 20)	High Zva (n = 16)	p-value
Age	79.2	76.4	0.82
BAV	4 (20%)	4 (25%)	0.72
SBP (mmHg)	132.4	149.8	0.026
Zva (mmHg/mL/m2)	3.62 ± 0.17	5.23 ± 0.77	0.006
AVA-TTE (cm2)	0.86 ± 0.09	0.86 ± 0.13	0.91
AVA-CMR (cm2)	0.78 ± 0.17	0.81 ± 0.07	0.295
LVMI-CMR (g)	82 ± 16.2	110 ± 18	0.034
LVEF-CMR (%)	66 ± 12.8	50 ± 8.2	0.047
LVEDV-CMR (mL)	127 ± 38.4	184 ± 28.4	0.03
RVEF-CMR (%)	59 ± 6.3	56 ± 10.4	0.1
RVEDV-CMR (mL)	108.2 ± 22.4	128.6 ± 22	0.042
LAVI-CMR (mL/m2)	65 ± 15.3	76 ± 12.5	0.044

